# Long-term safety and efficacy of open-label nabilone on sleep and pain in Parkinson´s Disease

**DOI:** 10.1038/s41531-024-00665-7

**Published:** 2024-03-15

**Authors:** Marina Peball, Beatrice Heim, Federico Carbone, Oliver Schorr, Mario Werkmann, Philipp Ellmerer, Kathrin Marini, Florian Krismer, Hans-Günther Knaus, Werner Poewe, Atbin Djamshidian, Klaus Seppi

**Affiliations:** 1 Department of Neurology, Medical University of Innsbruck, Innsbruck, Austria; 2 Department for Medical Genetics, Molecular, and Clinical Pharmacology, Medical University of Innsbruck, Innsbruck, Austria; 3 Department of Neurology, District Hospital of Kufstein, Kufstein, Austria

**Keywords:** Parkinson's disease, Parkinson's disease

## Abstract

The synthetic tetrahydrocannabinol-analog nabilone improved non-motor symptoms (NMS) in Parkinson’s disease (PD) patients in a placebo-controlled, double-blind, parallel-group, randomized withdrawal trial with enriched enrollment (NMS-Nab-study). This was a single-center open-label extension study to assess the long-term safety and efficacy of nabilone for NMS in PD. To be eligible for this study, patients had to be treatment responders during the previous NMS-Nab-trial and complete its double-blind phase without experiencing a drug-related serious/severe/moderate adverse event (AE). Patients were re-introduced to nabilone during an up-titration phase until their overall NMS burden improved. Nabilone was continued for six months with clinic visits every 3 months. Evaluation of AEs was based on self-report and clinical assessment. Twenty-two patients participated in the NMS-Nab2-study (age-median 68.33 y, 52% females, disease duration-median 7.42 y). Nabilone was well tolerated with concentration difficulties as the most common treatment-related AE (possibly/not related *n* = 1 each). One in two drop-outs discontinued because of an AE for which a prohibited concomitant medication needed to be introduced (night-time sleep problems). Efficacy evaluation showed a significant and lasting improvement in NMS burden according to the CGI-I (79% at V3). Nabilone improved overall sleep (NMSS Domain-2: –8.26 points; 95%CI –13.82 to –2.71; *p* = 0.004; ES = –0.72), night-time sleep problems (MDS-UPDRS-1.7: –1.42 points; 95 CI –2.16 to –0.68; *p* = 0.002; ES = –0.92), and overall pain (KPPS Total Score: –8.00 points; 95%CI –15.05 to –0.95; *p* = 0.046; ES –0.55 and MDS-UPDRS-1.9: –0.74 points; 95%CI –1.21 to –0.26; *p* = 0.008; ES = –0.74). This study demonstrates continuous long-term safety and efficacy in PD patients responding early to nabilone without intolerable side effects.

## Introduction

Parkinson’s disease (PD) is generally considered a neurological movement disorder as its diagnosis relies on the presence of typical motor symptoms^1^. The last decade has seen therapeutic advances targeting motor impairment^2^. However, it is widely known that PD patients also suffer from multiple non-motor symptoms (NMS). The latter significantly affects functionality in everyday life and quality of life^1^. NMS burden is a prominent topic raised by patients on clinic visits. Despite their significance, the spectrum of treatment options with a favorable risk/benefit balance for NMS in PD patients is still limited. This emphasizes the importance of exploration of further therapeutic options in adequate evidence-based clinical trials^3^.

Nabilone is a synthetic analog of tetrahydrocannabinol (THC), the psychoactive component of cannabis, with identical pharmacological properties^4,5^. It acts as a partial agonist on both cannabinoid 1 (CB1) and CB2 receptors in humans, thus mimicking the effects of THC but with the advantage of more predictable side effects and less euphoria^6,7^. We have recently performed a placebo-controlled, double-blind, parallel-group, enriched enrollment randomized withdrawal study (NMS-Nab Study) assessing the efficacy and safety of nabilone for NMS in PD patients^8^. Briefly, we found improved overall NMS burden (Movement Disorders Society—Unified Parkinson’s Disease-Rating Scale [MDS-UPDRS] Part I), especially reflected in the improvement of sleeping problems and anxiety^8,9^. The treatment was well tolerated. The NMS-Nab study was the first to assess the efficacy and safety of cannabinoids for the treatment of NMS in PD, making it a unique pilot trial. Prior uncontrolled studies evaluated the use of different cannabinoid applications in PD patients to find efficacy on problems with sleep, pain, anxiety, depression, or PD-associated psychosis^10–13^. Comparison is limited, however, due to the different routes of administration and substances (i.e., THC, cannabidiol). The endocannabinoid system (ECS) is believed to modulate neuronal circuits via the co-localization of cannabinoid 1 receptors with monoaminergic, gamma-aminobutyric acid-ergic, glutamatergic, and opioid synapses and neurons^14^. Data from animal and human PET studies show a high density of cannabinoid receptors in the basal ganglia believed to function as a regulator of dopamine release and uptake^8,15^. With regards to receptor distribution, the ECS is believed to both influence motor control as well as relevant non-motor circuits, e.g., for nociception, sleep regulation, or mood^5^. Combining clinical and pathophysiological information, the ECS is a promising target for the treatment of NMS in PD patients. To further examine the safety profile and treatment effects of nabilone, we performed an open-label extension study with participants of the NMS-Nab study. We aimed to demonstrate the long-term safety and efficacy of nabilone in PD patients.

## Results

**Fig. 1:**
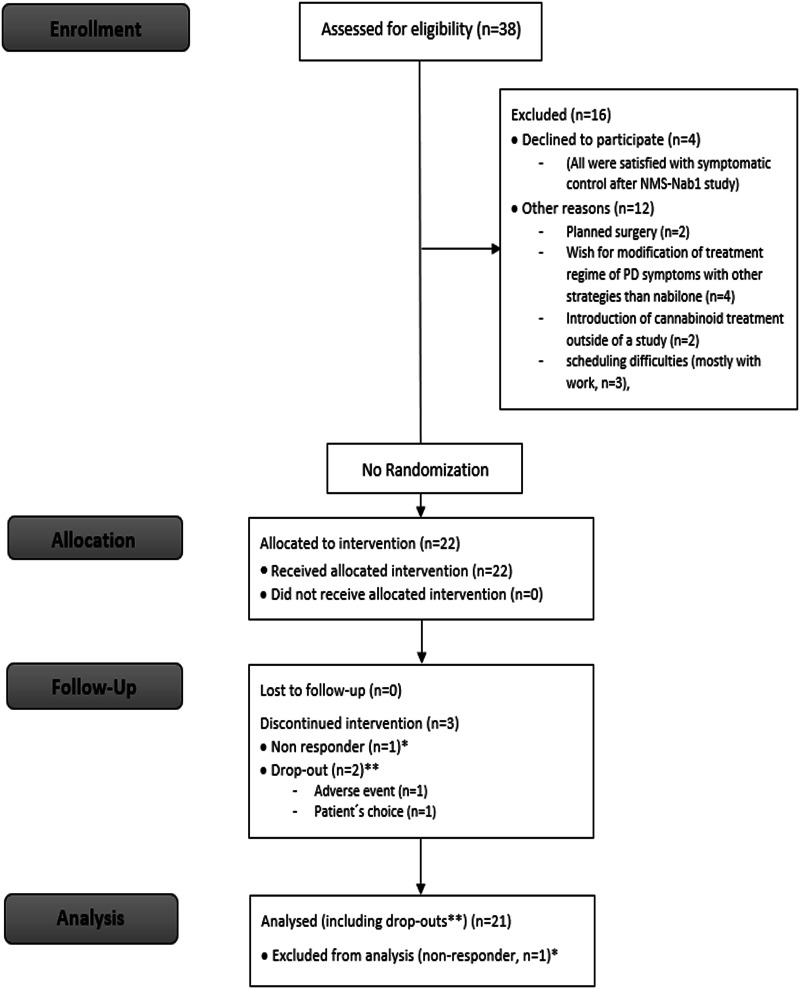
Flow Chart (adapted from CONSORT 2010). *The non-responder discontinued study participation before V1 and was therefore excluded from the analysis. **Data of regular visits were included up to the point of discontinuation (before V 2 in both drop-outs). n number.

All 38 patients who finished the double-blind phase of the preceding NMS-Nab study^8^ were assessed for eligibility. Four patients declined participation in the NMS-Nab2 study because they were satisfied with their symptomatic control at that time. In 12 patients, other reasons prevented them from taking part in this study: planned surgery (*n* = 2), wish for modification of treatment regime of PD symptoms with other treatment strategies than nabilone (*n* = 4), wish for introduction of cannabinoid treatment outside of a study (*n* = 2), scheduling difficulties (mostly with work, *n* = 3), other reason (*n* = 1; Fig. 1: Flow Chart).

Twenty-two patients participated in the NMS-Nab2 study between August 10, 2018, and January 31, 2020 (last patient last visit). There was no screening failure. Up-titration was started in all patients. One patient was a non-responder and therefore discontinued before V1. Two patients were drop-outs, one due to an adverse event (problems with night-time sleep) for which a prohibited concomitant medication (brotizolam) needed to be introduced. The other patient discontinued the therapy without consultation with the study team because of the deterioration of a pre-existing mild cognitive impairment (medical history). This did not resolve after nabilone discontinuation (1 patient of 21 = 4.76%). Nineteen patients finished the six months of continuous open-label nabilone treatment. Data from these 19 patients and of the two drop-outs (up to the point of discontinuation = before V 2) were included in the final analyses (Fig. 1: Flow chart).

The mean age of all participants at the screening was 67.23 ± 6.15 years (68.33) and the mean PD disease duration was 9.30 ± 6.04 years (7.42). Fifty-two % of participants were female (*n* = 11). The mean levodopa equivalent daily dose (LEDD) was 761 ± 637 (median: 500) mg at the Screening Visit as well as V1 (no change of LEDD). The mean daily nabilone dose after the titration phase (at V1) was 0.87 ± 0.44 mg (0.75 mg; Table 1). The mean LEDD at V3 was 667 ± 521 (median: 500) mg (*p* = 0.943). There was no change to relevant concomitant medication for non-motor symptoms during the six months of open-label treatment.

**Table 1:** Demographics at the screening visit (*n* = 21)

Age at SCR (years)	67.23 ± 6.15 (68.33, 62.21–71.25)
Daily nabilone dose (mg, at V1)	0.87 ± 0.44 (0.75) Range: 0.25–1.50
Females	11 (52.38%)
Disease duration (years)	9.30 ± 6.04 (7.42, 3.92–14.38)
Education (years)	12.81 ± 2.53 (12.00, 11.00–14.00)
Levodopa equivalent daily dose (in mg)	760.62 ± 637.36 (500.00)
Presence of disturbing dyskinesia	1 (4.76%)
Presence of motor fluctuations	10 (47.62%) All 1 point: Slight: ≤25% of waking day.

Data of continuous variables are presented as mean ± standard deviation (median, P25-P75).

Data of nominal variables are presented as *n* (%).

The presence of disturbing dyskinesia was defined as a score of ≥2 on item 4.2. and the presence of motor fluctuations was defined as a score ≥1 on item 4.3. of the MDS-UPDRS.

*SCR* screening, *v* visit. Higher score values indicate worse outcomes in all scales and questionnaires.

### Safety results

Common adverse events (AEs, >1 patient) are given in Table 2 and a full list of AEs and serious AEs (SAEs) is given in Supplementary Tables 1 and 2. Between V1 and V3, the most common adverse events were concentration difficulties (possibly related *n* = 2, not related *n* = 1). The daily dose of nabilone was slightly reduced in both patients where a relation to the study drug was deemed possible, which led to a resolution of the AEs. Suicidality according to the Columbia-Suicide Severity Rating Scale (C-SSRS) did not occur in any patient during the study and follow-up period.

**Table 2:** Safety analysis of the open-label phase

Most common AEs between V1 and V3 (*n* > 1)			
AE	Total (*n*)	Severity of AE (*n*)	
		Mild	Moderate
Respiratory tract infection	4	2	2
Concentration difficulties (disturbance in attention)	3	2	1
Intermittent falls	3	2	1
Urinary tract infection	2	2	0
Transient numbness of the face (hypoesthesia)	2	1	1
Osteopenia/Osteoporosis	2	1	1
Insomnia	2	0	2^a^
Lumbar pain (back pain)	2	0	2
Worsening of PD	2	0	2^b^
Arthrosis (osteoarthropathy)	2	0	2

*n* number, *AE* adverse event, *SAE* serious adverse event, *PD* Parkinson´s Disease.

Definitions: Mild: i.e., Discomfort noticed but no disruption of normal daily activity. Moderate: i.e., Discomfort sufficient to reduce or affect daily activity; no treatment or medical intervention is indicated although this could improve the overall well-being or symptoms of the patient.

^a^Leading to discontinuation in one patient.

^b^1 SAE.

No participant died during the study. There was no SUSAR during this trial. There were five SAEs in four patients reported during the conduct of this trial, all of which were rated “unrelated to nabilone” by the investigators (hospitalization due to nausea and vomiting after the introduction of levodopa treatment in one patient during titration phase; hospitalization due to worsening of PD for implementation of intrajejunal levodopa treatment (pump system) for symptomatic relief in one patient during the open-label phase; diagnosis of an adenocarcinoma of the rectum for which the patient received surgical treatment as well as combined radiochemotherapy; hospitalization for surgical decompression of a disc protrusion in the lumbar segment 4/5 in one patient with lumbar pain during open-label treatment with nabilone).

There was a significant difference in systolic blood pressure between the supine position and after three minutes of standing at the Screening Visit (5.53 ± 12.14 (median: 7.00); *p* = 0.038), the Visit V1 (4.95 ± 6.36 (5.00); *p* = 0.005), and the Visit V3 (6.05 ± 11.40 (8.00); *p* = 0.022). There was no significant difference between the mean score of the hallucination–(item 1.2; *p* = 0.366), day-time sleepiness—(item 1.8; *p* = 0.803), and orthostatic hypotension (OH)—(item 1.12; *p* = 1.000) items of the MDS-UPDRS between the Screening Visit and V3.

The mean Mini-Mental State Exam (MMSE) at the Screening Visit of the double-blind NMS-Nab study was 29.37 ± 0.96 (median: 30.00) and at V3 of the open-label study 28.95 ± 1.31 (median: 29.00) points (*n* = 19). The Montreal Cognitive Assessment (MoCA) score was 28.11 ± 1.29 (median: 28.00) at the Screening Visit of the previous NMS-Nab study and 28.21 ± 1.90 (median: 29.00) points at V3 after the open-label phase of the NMS-Nab2 study (*n* = 19). The mean change of the MMSE between the Screening Visit of the NMS-Nab1 Study and the V3 Visit of this study was 0.42 ± 1.84 points (95% CI –0.46 to 1.31; *p* = 0.298; ES = 0.23) and of the MoCA –0.11 ± 1.94 points (95% CI –1.04 to 0.83; *p* = 0.775; ES = –0.05).

### Efficacy results

**Fig. 2:**
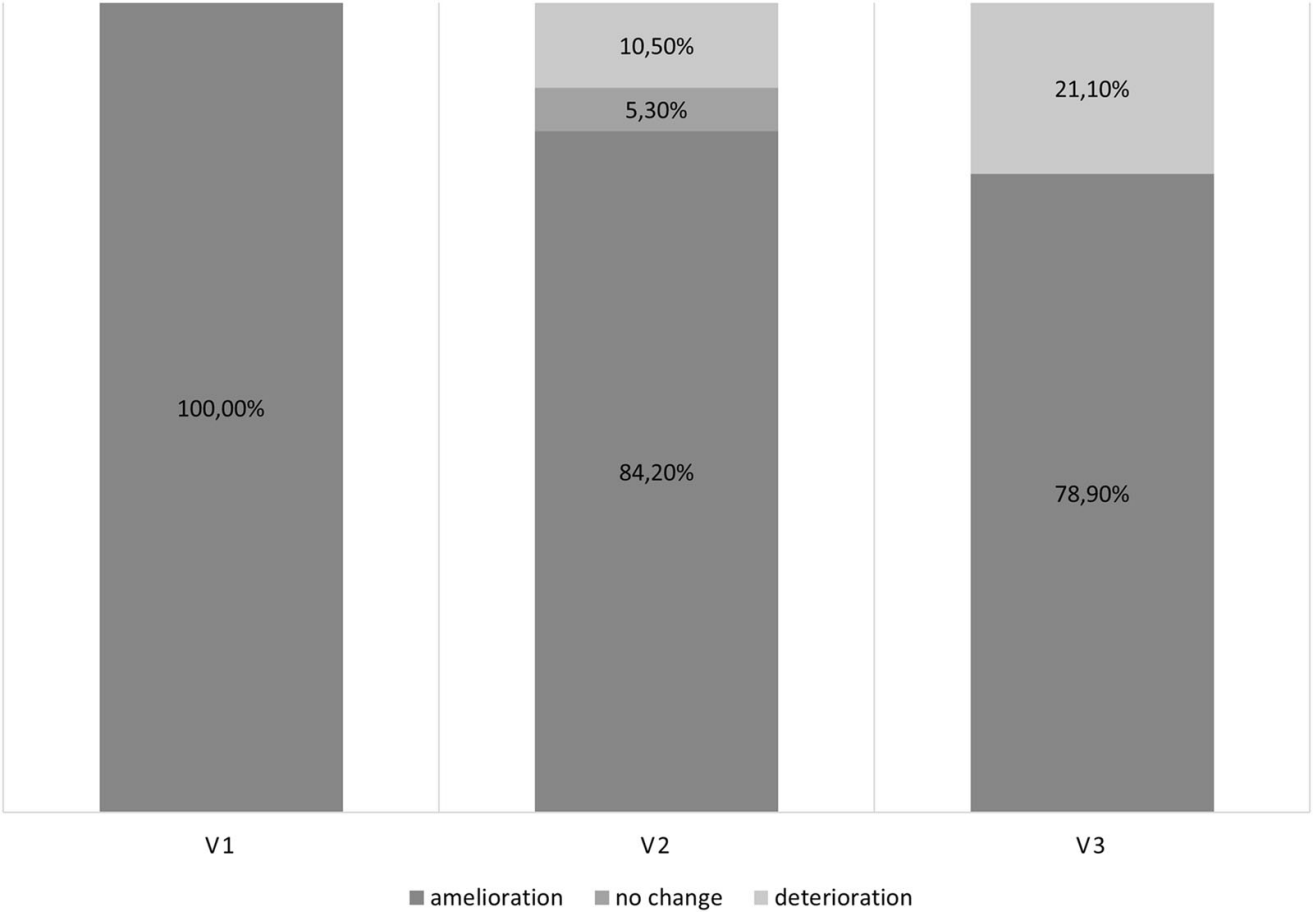
Clinical Global Impression—Global Improvement Scale during the study. CGI difference between V1 and V3: *p* = 0.002, *φ* coefficient = 0.034.

**Fig. 3:**
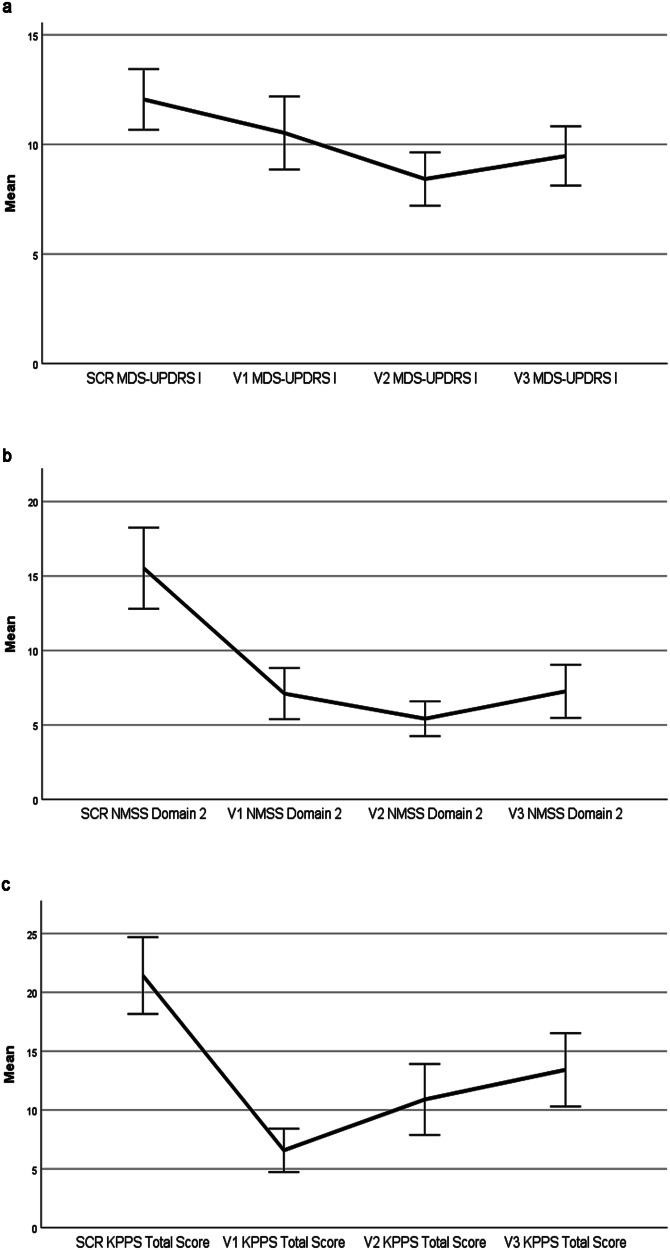
Change of endpoint scores during the open-label administration of nabilone. SCR screening, V visit. Error bars: +/– 1 SE (standard error of the mean). **a** Change of MDS-UPDRS I during the open-label administration of nabilone. MDS-UPDRS Movement Disorder Society–Unified Parkinson´s Disease-Rating Scale. **b** Change of the NMSS Domain 2 during the open-label administration of nabilone. NMSS Non-Motor Symptoms Scale. NMSS Domain 2: Sleep/Fatigue. **c** Change of the KPPS during the open-label administration of nabilone. KPPS, King’s Parkinson´s Disease Pain Scale.

**Fig. 4:**
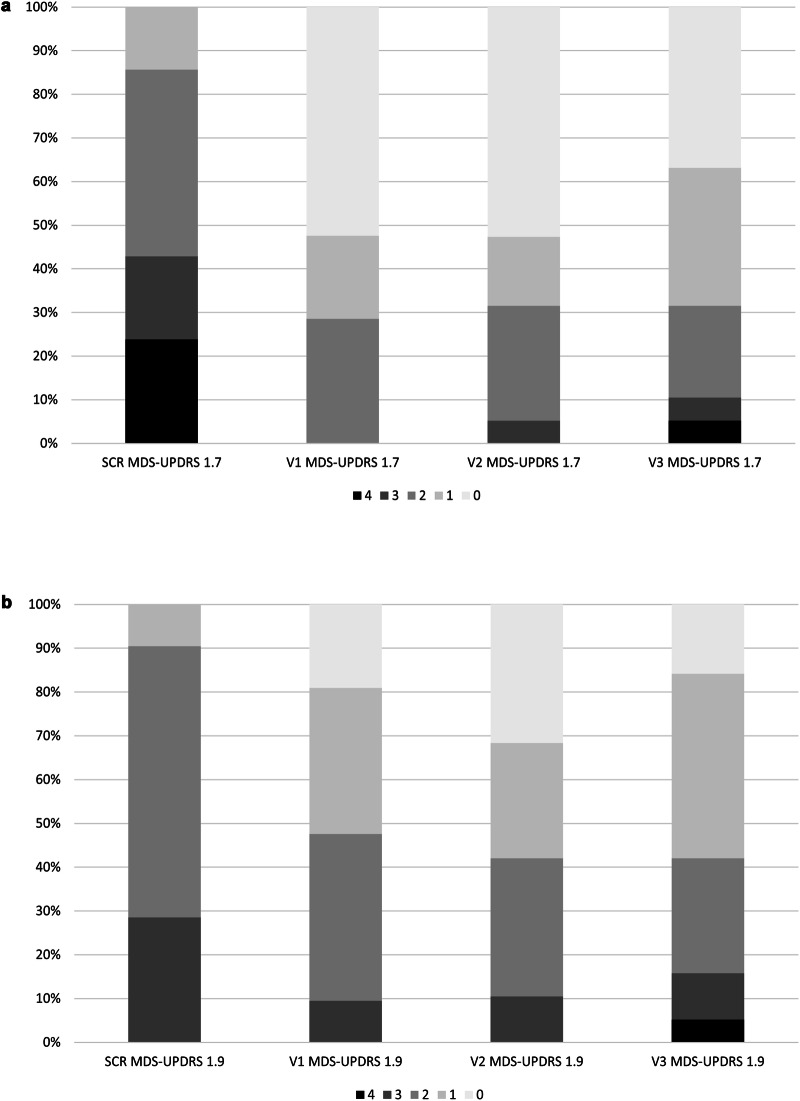
Change of MDS-UPDRS item scores during the open-label administration of nabilone. SCR screening, V visit, MDS-UPDRS Movement Disorder Society–Unified Parkinson´s Disease-Rating Scale. **a** Change of MDS-UPDRS item 1.7 during the open-label administration of nabilone. MDS-UPDRS-1.7: Night-time sleep problems. **b** Change of the MDS-UPDRS 1.9 during the open-label administration of nabilone. MDS-UPDRS-1.9: Pain and other sensations.

The results of the efficacy analyses are displayed in Table 3, Figs. 2, 3a–c, and 4a, b, as well as Supplementary Tables 3 and 4. There was no significant difference between the assessed scales and questionnaires from V1 to V3 (all *p* > 0.073), except for the Hospital Anxiety and Depression Scale-Depression Subscale (HADS-D) with a mean difference of –1.00 ± 2.08 points (95% CI –2.00 to 0.00; *p* = 0.044; ES = 0.48; Supplementary Table 3). The Clinical Global Impression of Improvement Scale (CGI-I) displayed improvement of NMS burden in all patients at V1 (as per responder criterion). At V3, still, 78.90% of the patients reported an amelioration, while 21.10% rated their burden of NMS deteriorated in the CGI-I compared to screening (Fig. 2).

**Table 3:** Change in efficacy endpoint scores between screening and V3, patients *n* = 19

	SCR *n* = 21	V3 *n* = 19	Mean change (95% CI) between SCR visit and V3	*p*-value	Effect size
Secondary efficacy endpoints					
MDS-UPDRS-I	12.05 ± 5.79 (11.00) (8.50–14.00)	9.47 ± 5.90 (8.00) (4.00–14.00)	–2.58 (–5.00; –0.16)	0.052	−0.51
MDS-UPDRS Motor Sum Score II + III	41.24 ± 16.88 (43.00) (27.00–52.00)	40.47 ± 15.36 (37.00) (30.00–46.00)	0.68 (–3.48; 4.84)	0.763	0.08
MDS-UPDRS-III	28.95 ± 11.12 (33.00) (18.50–38.50)	29.37 ± 10.10 (28.00) (21.00–38.00)	1.21 (–1.85; 4.27)	0.513	0.19
MDS-UPDRS item 1.4^a^	0.90 ± 1.09 (0.00) (0.00–2.00)	0.58 ± 0.77 (0.00) (0.00–1.00)	–0.37 (–0.91; 0.17)	0.158	–0.33
MDS-UPDRS item 1.7^a^	2.52 ± 1.03 (2.00) (2.00–3.50)	1.11 ± 1.15 (1.00) (0.00–2.00)	–1.42 (–2.16; –0.68)	0.002	−0.92
NMSS Total Score	48.62 ± 31.10 (42.00) (20.50–74.50)	38.95 ± 25.82 (29.00) (18.00–62.00)	–7.68 (–18.96; 3.59)	0.205	–0.33
NMSS Domain 2	15.48 ± 11.38 (12.00) (6.00–21.00)	7.26 ± 7.77 (5.00) (2.00–8.00)	–8.26 (–13.82; –2.71)	0.004	−0.72
Exploratory efficacy endpoints					
MDS-UPDRS-II	12.29 ± 7.21 (12.00) (6.00–15.50)	11.11 ± 6.82 (8.00) (6.00–16.00)	–0.53 (–2.25; 1.20)	0.958	–0.15
MDS-UPDRS Total Score	55.81 ± 22.45 (55.00) (38.00–69.50)	52.58 ± 22.45 (50.00) (37.00–58.00)	–1.47 (–6.99; 4.04)	0.376	–0.13
MDS-UPDRS item 1.8^a^	1.05 ± 0.74 (1.00) (0.50–2.00)	1.05 ± 0.91 (1.00) (0.00–2.00)	0.05 (–0.39; 0.49)	0.803	0.06
MDS-UPDRS item 1.9^a^	2.19 ± 0.60 (2.00) (2.00–3.00)	1.47 ± 1.07 (1.00) (1.00–2.00)	-0.74 (–1.21; –0.26)	0.008	−0.74
H&Y	2.05 ± 0.38 (2.00) (2.00–2.00)	2.11 ± 0.32 (2.00) (2.00–2.00)	0.05 (–0.20; 0.31)	0.655	0.10
NMSS Domain 3	6.95 ± 7.86 (3.00) (1.50–10.50)	6.89 ± 8.23 (4.00) (1.00–9.00)	0.79 (–3.80; 5.38)	0.887	0.08
NMSS Domain 4	0.71 ± 2.03 (0.00) (0.00–0.00)	0.16 ± 0.50 (0.00) (0.00–0.00)	-0.63 (–1.60; 0.34)	0.180	–0.32
NMSS Domain 5	3.86 ± 6.73 (1.00) (0.00–5.00)	3.68 ± 6.08 (2.00) (0.00–6.00)	0.53 (–0.56; 1.61)	0.381	0.23
NMSS Domain 6	3.14 ± 3.77 (2.00) (0.00–5.50)	3.74 ± 4.57 (2.00) (0.00–8.00)	0.68 (–1.39; 2.76)	0.624	0.16
NMSS Domain 7	6.67 ± 6.10 (5.00) (1.00–12.00)	6.74 ± 5.76 (6.00) (2.00–13.00)	-0.11 (–2.50; 2.29)	0.721	–0.02
NMSS Domain 8	0.00 ± 0.00 (0.00) (0.00–0.00)	0.42 ± 1.12 (0.00) (0.00–0.00)	0.42 (–0.12; 0.96)	0.109	0.38
NMSS Domain 9	10.52 ± 8.01 (8.00) (6.00–12.00)	8.21 ± 5.90 (8.00) (3.00–12.00)	–1.74 (–5.21; 1.73)	0.279	–0.24
KPPS Total Score	20.86 ± 13.66 (17.00) (9.50–32.00)	13.42 ± 13.60 (8.00) (1.00–24.00)	–8.00 (–15.05; -0.95)	0.046	−0.55
HADS-A	5.38 ± 4.91 (5.00) (1.00–8.50)	4.68 ± 3.74 (4.00) (2.00–9.00)	–0.74 (–1.81; 0.34)	0.216	–0.33
HADS-D	4.90 ± 3.74 (4.00) (2.00–8.00)	4.42 ± 3.20 (4.00) (2.00–7.00)	–0.63 (–1.64; 0.38)	0.295	–0.30
PDQ-8 SI	54.02 ± 18.60 (56.25) (37.50–75.00)	54.11 ± 16.44 (56.25) (40.63–68.75)	0.00 (–3.82; 3.82)	0.886	0.00
ESS	6.95 ± 4.46 (6.00) (5.00–9.00)	7.32 ± 4.58 (7.00) (4.00–8.00)	0.47 (–0.70; 1.65)	0.456	0.20
FSS	32.10 ± 15.08 (29.00) (21.50–43.50)	29.89 ± 14.35 (27.00) (19.00–39.00)	–0.63 (–5.26; 3.99)	0.856	–0.07
QUIP-RS	0.14 ± 0.66 (0.00) (0.00–0.00)	0.37 ± 1.12 (0.00) (0.00–0.00)	0.37 (–0.17; 0.91)	0.180	0.33

Data of continuous variables are presented as mean ± standard deviation (median, P25-P75).

*CI* confidence interval, *MDS-UPDRS* Movement Disorder Society–Unified Parkinson´s Disease-Rating Scale, *NMSS* Non-Motor Symptoms Scale, *KPPS* King’s Parkinson´s Disease Pain Scale, *HADS-A/-D* Hospital Anxiety and Depression Scale-Anxiety/-Depression, PDQ-8 SI Parkinson´s Disease Questionnaire–8 Summary Index, ESS Epworth Sleepiness Scale, FSS Fatigue Severity Scale, QUIP-RS Questionnaire for Impulsive-Compulsive Disorders in Parkinson’s Disease–Rating Scale, SCR screening, V visit. Higher Score values indicate worse outcomes in all scales and questionnaires.

NMSS Domains: Domain 1: Cardiovascular, Domain 2: Sleep/Fatigue, Domain 3: Mood/Apathy, Domain 4: Perceptual problems/Hallucinations, Domain 5: Attention/Memory. Domain 6, Domain 7: Urinary, Domain 8: Sexual dysfunction, Domain 9: Miscellaneous.

For all *p*-values, the significance level was set at *p* ≤ 0.05. For the secondary efficacy endpoints: *p*-values corrected for multiple testing (Bonferroni correction) with *p*-value cut-off set at *p* < 0.007 (0.05/7).

Effect size according to Cohen´s *D*. Cohen´s *D* of 0.2, 0.5, and 0.8 were considered ‘small’, ‘medium’, and ‘large’ effect sizes.

^a^MDS-UPDRS 1.4: Anxious mood, MDS-UPDRS-1.7: Night-time sleep problems, 1.8: Day-time sleepiness, 1.9: Pain and other sensations.

Mean change of the MDS-UPDRS-I score from screening to V3 was –2.58 points (95% CI –5.00 to –0.16, *p* = 0.052) with a moderate effect size (–0.51; Fig. 3a). Mean change of item 1.7 of the MDS-UPDRS-I (i.e., Night-time sleep problems) showed a significant difference of –1.42 points (95% CI –2.16 to –0.68; *p* = 0.002) with a large ES of -0.92 (Fig. 4a). In line with this, the score of Domain 2 of the NMS-Scale (NMSS) (i.e., Sleep/Fatigue) significantly decreased by –8.26 points (95% CI –13.82 to –2.71; *p* = 0.004; ES = –0.72) from screening to V3 (Fig. 3b). Additionally, the King’s PD Pain Scale (KPPS) Total score was significantly reduced by -8.00 points (95% CI –15.05 to –0.95; *p* = 0.046; ES = –0.55) between screening and V3 (Fig. 3c). Reflecting these findings, item 1.9 of the MDS-UPDRS-I (i.e., Pain and other sensations) also significantly decreased with nabilone treatment with a mean change of –0.74 points (95% CI –1.21 to –0.26; *p* = 0.008) and a moderate ES (–0.74, Fig. 4b). There was no significant difference between screening- and V3- score values in the MDS-UPDRS Parts II, III, and motor sum score (all *p* > 0.513), the MDS-UPDRS item 1.4 (*p* = 0.158), the other NMSS Domains and the NMSS Total Score (all *p* > 0.109), the Hoehn and Yahr scale (*p* = 0.655), the HADS-Anxiety Subscale (HADS-A; *p* = 0.216) and -D (*p* = 0.295) scores, the PD-Questionnaire-8 (PDQ-8) summary index (*p* = 0.886), the Epworth Sleepiness Scale (ESS; *p* = 0.456), the Fatigue Severity Scale (FSS; *p* = 0.856), and the Questionnaire for Impulsive-Compulsive Disorders in PD-Rating Scale (QUIP-RS; *p* = 0.180; Table 3).

Of the cohort at screening, 11 patients were in the former nabilone and 10 in the former placebo group. One patient in each group was a drop-out before V3. There was no significant difference between the former nabilone and placebo groups in any of the relevant efficacy outcome measures (all *p* > 0.427; Supplementary Table 4).

## Discussion

The NMS-Nab2 study represents an open-label follow-up study of the preceding randomized placebo-controlled, double-blind, parallel-group, enriched enrollment randomized withdrawal NMS-Nab trial^8^. We examined the long-term safety and efficacy of treatment with the THC-analog nabilone in PD patients.

Generally, nabilone was well tolerated during the 6-months of open-label use. Moreover, the NMS-Nab2 study suggests improvement of non-motor symptom burden (according to the CGI-I), sleep problems, and pain.

Most AEs in this trial were not related to nabilone and were expected in a long-term follow-up study (e.g., infections, intermittent falls). The most common treatment-related AE was difficulty with concentration, which was resolved in both patients after a decrease in the nabilone dose. Concentration difficulties are known as possible side effects from the Summary of Product Characteristics (SmPC) and have been described in other controlled trials using nabilone^16–18^. Trials of acute cannabis consumption in healthy volunteers show inconsistent results regarding cognitive function. Deficits in verbal learning/memory and spatial working memory were most prominently reported^19,20^. Importantly in our study, there was no continuous overall cognitive decline associated with long-term nabilone use as the patient´s MMSE and MoCA scores were not different than before their first-ever nabilone use (i.e., before the previous NMS-Nab study). It must be noted, that total MMSE and MoCA scores do not necessarily have to reflect mild changes in working memory (i.e., attention), learning, or memory attributable to cannabinoid intake. However, very few patients reported concentration difficulties in this study cohort. Moreover, we have previously performed an eye-tracking evaluation (using the Tobii TX-300 eye tracker and the Tobii Pro Lab Software v.1.83) in a subset of participants of the double-blind NMS-Nab study, comparing saccadic paradigms between the Screening visit and the end of the double-blind treatment phase. Nabilone use did not impair top-down inhibitory control that requires intact frontal networks, visual fixation, saccadic control, or learning (error rates reduction)^21^. Integrating this evidence and taking into consideration the neurodegenerative nature of PD and already associated changes in cognitive function, our results emphasize the good overall tolerability of nabilone with preserved cognition in this patient group.

A recent meta-analysis of randomized, double-blind, placebo-controlled studies using nabilone (*n* = 6) found the most common AEs to be drowsiness, dizziness, headache, and dry mouth^18^. Interestingly, postural dizziness was not commonly reported by the patients during continuous nabilone use in this study. Measured orthostatic systolic blood pressure decrease did not qualify for the diagnosis of OH and no respective symptoms were noted by the patients during test performance. This may represent tolerance to the study drug´s side effects over time. OH does not seem to deter the introduction of cannabinoid treatment in PD patients.

Safety data on the long-term use of cannabinoids is lacking from high-quality trials and most trials do not have an appropriate sample size. This hinders an unbiased assessment of the safety profile of cannabinoids^18^. Our study complements this information gap with additional strengths of a thorough assessment of safety and tolerability with frequent patient contact and a long follow-up period compared to other clinical trials using nabilone^18^. Treatments recommended by guidelines for non-motor symptoms in PD patients (i.e., selective serotonin-norepinephrine reuptake inhibitors for depression or hypnotics for sleep disorders) may have a more adverse side effect profile than what we learned from the use of nabilone in this study^3^. However, no trial has ever directly compared safety and tolerability.

The secondary endpoints in this study assessed efficacy. It must be emphasized, that the NMS-Nab2 study included treatment responders, i.e., patients with improvement of NMS with nabilone treatment, that did not experience AEs causing them to drop-out of the previous trial. Thus, the positive results of the efficacy analysis reflect a long-lasting benefit from cannabinoids in those patients who initially respond to the medication without intolerable side effects. Long-term open-label treatment with nabilone improved overall non-motor symptom burden as demonstrated by the CGI-I (79% improved until V3) and the MDS-UPDRS-I score (-2.58 points from screening to V3, p = 0.052, ES = –0.51). This is in line with our findings from the preceding NMS-Nab study^8^. Nabilone also proved to ameliorate overall sleep (NMSS Domain 2, *p* = 0.004, ES = –0.72) and night-time sleep problems (MDS-UPDRS item 1.7, *p* = 0.002, ES = –0.92) in PD patients. A post hoc analysis of our first trial already demonstrated a significant improvement in sleep measurements in PD patients treated with nabilone compared to the placebo group^9^. Sleep problems in PD are complex and often multifactorial including primary dysfunction of sleep regulation due to neurodegeneration as well as secondary causes. The latter include awakening due to immobility while turning in bed, unwanted effects of PD medication on sleep, or influence of comorbidities such as restless legs syndrome (RLS) or urinary problems^3^. The ECS is known to be involved in the regulation of the circadian sleep-wake cycle and promotion as well as maintenance of sleep^9,22^. Cannabidiol has been used in PD patients before with inconsistent results^13,23,24^. Nabilone however has not been thoroughly assessed for sleep in PD patients before the NMS-Nab trials. Clinical trial data from other cohorts suggests that nabilone improves nightmares and sleep duration in posttraumatic stress disorder as well as sleep quality in fibromyalgia patients with chronic pain^22^. The association of chronic pain with sleep disturbances is inevitable.

Cannabinoid treatment has long been studied for its use in chronic pain. CB1 receptors are expressed in brain regions involved in the perception of pain such as the thalamus and amygdala but also in regions that modulate nociception such as midbrain periaqueductal gray matter. Moreover, the dorsolateral funiculus and superficial dorsal horn of the spinal cord have a large density of CB1 receptors, mostly in interneurons. Lastly, CB1 receptors are present in nerve terminals of peripheral sensory neurons (via axonal transport from the dorsal root ganglia). It is believed that the ECS operates via modulation of neurotransmitter release on a peripheral and central (spinal and supraspinal) level, including glutamate, glycine, and GABA^14^. Additional non-neuronal effects such as inhibition of release of pro-inflammatory cytokines or activation of CB2 receptors on cells of the immune system may also contribute to pain modulation^14^. This implies the possibility of the efficacy of cannabinoids in various types of pain (i.e., inflammatory pain, neuropathic pain). Nabilon has been shown to improve chronic pain, neuropathic pain (i.e., in patients with diabetes), pain in fibromyalgia, and pain associated with spasticity (i.e., in multiple sclerosis)^17,25,26^. The overall evidence however is limited with quality ratings of most studies (GRADE) being low^27^. In our study, overall pain was significantly reduced by treatment with nabilone (KPPS Total Score, *p* = 0.046, ES = –0.55 and MDS-UPDRS item 1.9, *p* = 0.008, ES = –0.74), reflecting the nociceptive modulation properties of the ECS. Open-label treatment with nabilone has also shown a positive effect on pain in our previous study^8^. Double-blind treatment did not reveal a difference from the placebo group. Thus, a placebo effect may possibly influence the current result. However, this study longitudinally demonstrated an effect of nabilone on pain in PD patients.

Our study has some limitations to consider. The small sample size, derived from the inclusion of patients previously participating in the NMS-Nab study, limits the generalizability of the results. Moreover, the burden of pain or anxiety was an inclusion criterion in the double-blind placebo-controlled NMS-Nab study (respective MDS-UPDRS I item of ≥2 points) and may thus be overrepresented in this patient cohort. However, the well-defined cohort, thorough safety assessment, long follow-up period, and usage of standardized outcome measures are notable strengths of this study.

In conclusion, nabilone was well tolerated in our long-term open-label study of PD patients. Additionally, it improved overall non-motor symptom burden, sleep problems, as well as pain. With respect to our previous randomized placebo-controlled, double-blind, parallel-group, enriched enrollment randomized withdrawal NMS-Nab trial, the efficacy results suggest that most PD patients benefitting from cannabinoids continue to be treatment responders even after six months. Our data suggests that nabilone is a promising and safe treatment agent for reducing non-motor symptom burden in PD patients.

## Methods

This was a single-center open-label extension study for participants of the preceding placebo-controlled, double-blind, parallel-group, enriched enrollment randomized withdrawal trial (i.e., NMS-Nab Study)^8^. The study aimed to assess the long-term safety and efficacy of treatment with nabilone in PD patients.

The diagnosis of PD was based on standard criteria and NMS severity was assessed by the non-motor section (Part I) of the MDS-UPDRS. In order to be eligible for this study, patients had to be treatment responders during the titration phase of the NMS-Nab trial^8^ and have completed the double-blind phase of it without experiencing a drug-related serious adverse event (SAE) or drug-related moderate or severe adverse event (AE). Treatment responders were defined as patients rating their NMS as “much improved” or “very much improved” on the 7-point Clinical Global Impression of Improvement Scale (CGI-I) during the introduction and titration of nabilone. The NMS-Nab trial excluded patients with inadequately controlled motor complications (i.e., a score ≥2 on one of the items of the MDS-UPDRS Part IV at screening), reflected in this study´s population.

### Ethical statement

The study was approved by the local ethics committee and the Austrian national regulatory authorities. All individuals gave written informed consent before participation. No participant received a stipend. All procedures were performed in accordance with the 1964 Declaration of Helsinki and its later amendments.

### Procedures

**Fig. 5:**
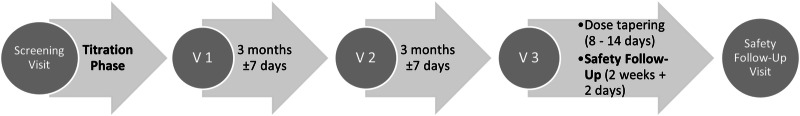
Schedule of trial activities. The mean duration of the titration phase was 30 ± 12 days (median: 27 days) and of the treatment phase (i.e., open-label nabilone administration between V1 and V3) 179 ± 7 days (median: 179 days).

The preceding randomized, double-blind NMS-Nab study ended with tapering of the investigational drug (i.e., nabilone or placebo) in all patients after the double-blind treatment phase. A safety follow-up visit was performed after 2 weeks of discontinuation from the study drug. The Screening Visit was ideally performed on the same day or scheduled. At the Screening Visit, oral nabilone was re-introduced in all patients with the same titration process as it was performed in the preceding NMS-Nab trial. Nabilone was given daily starting with a dose of 0.25 milligrams (mg, 1 capsule) in the evening after the Screening Visit and concluded with twice daily dosing. During regular telephone calls with the study team, the nabilone dose could be increased to 0.25 mg-steps every 1 to 4 days, optimally up to the dose the patient had in the open-label titration phase of the preceding NMS-Nab study. If necessary, modification of this dose was possible upon the investigator´s decision. Dose adjustments were performed until patients again met the responder criterion (CGI-I). Patients failing to meet this criterion at the maximum daily dose of 2 mg or patients with intolerable AEs related to nabilone were discontinued. The titration phase lasted up to 28 days and responders proceeded into an open-label treatment period of six months on a stable nabilone dose following a study visit (V1). Dose adjustments were only possible after consultation with the study team and if the CGI-I and thus the NMS-control deteriorated. Patients were able to continue as per protocol thereafter. Visits were performed every three months (V 2, V3). The open-label phase ended with a termination visit (V3) from which on the study drug was tapered in all patients in 0.25 mg-decrements. During tapering, the patients received phone calls every other day. A Safety Telephone Call and a Safety Follow-Up Visit were performed 5 days ± 2 days and 2 weeks + 2 days after the last intake of study drug. In summary, this study comprised five on-site study visits and regular telephone calls during titration phases (Fig. 5, NMS-Nab2 study). Safety parameters were evaluated throughout the study via telephone calls and at on-site visits with reference to the number of subjects (%) who discontinued the study due to an AE or other reasons, AEs, clinical examination findings, vital signs including orthostatic hypotension (OH), the Columbia-Suicide Severity Rating Scale (C-SSRS) as well as the hallucination- (1.2), day-time sleepiness- (1.8), and OH- (1.12) items of the MDS-UPDRS. Blood pressure was measured with the patient in the supine position (after having been in this position for 10 minutes) and after 3 minutes in the standing position after the postural change. Clinical assessments included the MDS-UPDRS, NMS-Scale (NMSS), Hospital Anxiety and Depression Scale (HADS), PD-Questionnaire-8 (PDQ-8), Mini-Mental State Exam (MMSE), Montreal Cognitive Assessment (MoCA), Epworth Sleepiness Scale (ESS), Fatigue Severity Scale (FSS), King’s PD Pain Scale (KPPS), and Questionnaire for Impulsive-Compulsive Disorders in PD-Rating Scale (QUIP-RS). CGI-I ratings were evaluated at V1, V 2, and V3. Because the double-blind NMS-Nab study was ongoing, patients and outcome assessors were still blinded to the initial treatment assignment. The levodopa equivalent daily dose (LEDD) was calculated using published conversion factors^28^. The safety data monitoring board (H.G.K., K.S., and M.P.) supervised safety parameters during study conduction.

### Statistical analysis

Sample size considerations derive from calculations of the previous NMS-Nab study^8^. Thus, up to 48 subjects who completed the preceding randomized placebo-controlled, double-blind, parallel-group, enriched enrollment randomized withdrawal study (NMS-Nab Study) and met patient exclusion/inclusion criteria were able to be enrolled in this open-label extension study.

Safety and tolerability summaries were based on a safety set which included all patients receiving at least one dose of study medication and completing at least V1. The efficacy analyses included all screened subjects with at least one visit after screening (i.e., treatment responders). Data of drop-outs after V1 is included up to the point of study discontinuation.

The study´s primary endpoint addressed long-term safety and tolerability. The safety analysis was a descriptive analysis of all the above-mentioned events and tolerability issues occurring throughout the overall course of the study.

The secondary efficacy endpoints evaluated the long-term efficacy of nabilone between V1 and V3 using a Wilcoxon matched-pairs test. Additionally, the change from screening (i.e., before study drug intake) to V3 (i.e., end of six months open-label treatment phase) was assessed via Wilcoxon matched-pairs test as a mean change of the above-mentioned scales. For this analysis, efficacy values were divided into “secondary” (i.e., significant difference in the NMS-Nab study; MDS-UPDRS-I score, MDS-UPDRS Motor Sum Score (Parts II + III), MDS-UPDRS-III score, MDS-UPDRS item 1.7, MDS-UPDRS item 1.4, NMSS Total Score, NMSS Domain 2 score) and “exploratory” (all other values) based on the results of the NMS-Nab study. Bonferroni correction for multiple comparisons was performed for the former. The mean change of the items 1.4 (i.e., Anxious mood), 1.7 (i.e., Night-time sleep problems), 1.8 (i.e., Day-time sleepiness), and 1.9 (i.e., Pain and other sensations) of the MDS-UPDRS-I between screening and V3 was also evaluated using a Wilcoxon matched-pairs test. The CGI-I was compared between V1 and V3. The score values of the MMSE and MoCA of the Screening Visit of the preceding randomized placebo-controlled, double-blind NMS-Nab study (i.e., before the first-ever intake of nabilone) were compared to scores from the V3 Visit of this study to assess a long-term change in cognitive function. As a post hoc analysis, a Mann–Whitney *U*–test was used to assess whether there is a difference in the mean change of relevant outcome parameters from screening to V3 between the former nabilone and placebo groups. Effect sizes (ES) for the different endpoints were calculated according to Cohen’s D^29^, except for the CGI-I where φ coefficient was used^30,31^. Cohen’s D of 0.2, 0.5, and 0.8 as well as φ coefficient of 0.1, 0.3, and 0.5 were considered a “small,” “medium,” and “large” effect size.

Since an interpolation of data was not performed in case of a drop-out, the primary analysis is a per-protocol analysis. No interim analysis was planned and performed.

For all analyses, statistical significance was set at the 2-sided 5% α-level. SPSS version 25.0 for Windows (SPSS, IBM Corporation, and other(s) 1989, 2017, Chicago, IL) was used to analyze data. This trial is registered with ClinicalTrials.gov (NCT03773796) and EudraCT (2017-004253-16).

### Reporting summary

Further information on research design is available in the Nature Research Reporting Summary linked to this article.

### Supplementary information

Reporting Summary

Supplementary Files

## Data Availability

Data that support the findings of this study are available from the first or corresponding author upon request and fulfilling data sharing regulations approved by the local ethics committee. Only deidentified individual data that underlie the results reported in this manuscript will be made available. Proposals should be directed to the first or corresponding author. Data will be available solely for the purpose of achieving the aims in the approved proposal. Data will only be shared via individual secured network connections.
