# Supplementary material for: Long-term safety and efficacy of open-label nabilone on sleep and pain in Parkinson´s Disease
Source: NPJ Parkinsons Dis. 2024 Mar 15;10:61. doi: 10.1038/s41531-024-00665-7 (PMC10943069; doi:10.1038/s41531-024-00665-7)
Supplement: Supplementary file 2 — Supplementary Files [file 41531_2024_665_MOESM2_ESM.pdf]

Supplementary Files

Supplementary Table 1: Safety Analysis of the titration phase: Full list of AEs and SAEs

Titration phase
**AEs (n=13)**
**Mild**<br>Vertigo (n=2): possibly related (n=1), probably related (n=1)<br>Pain (n=2): possibly related (n=1, in both legs), not related (n=1, diffuse)<br>Headache (n=1): possibly related<br>Inner tension (n=1): possibly related<br>Sleep disturbances (maintenance of sleep, n=1): not related<br>Upper respiratory tract infection (n=1): not related<br>Dry skin (n=1): not related<br>Fall (n=1): not related<br><br>**Moderate**<br>Frontal sinusitis (n=1): not related<br>Insomnia (n=1): not related
**SAEs (n=2)**
**Moderate:**<br>Medication-induced Nausea (n=1): not related, hospitalization<br>Medication-induced Emesis (n=1): not related, hospitalization

Abbreviations: n, number; AE, adverse event; SAE, serious adverse event.

Supplementary Table 2: Safety Analysis of the open-label phase: Full list of AEs and SAEs

**Open-label phase [a]**
**AEs (n=39)**
**Mild**
Concentration difficulties (n=2): possibly related (n=1), not related (n=1)
Intermittent falls (n=2): not related
Upper respiratory tract infection (n=2): not related (viral n=1, bacterial n=1)
Urinary tract infection (n=2): not related
Vertigo (n=1): possibly related
Muscle cramps (n=1): not related
Hypercholesterinaemia (n=1): not related
Osteoporosis (n=1): not related
Haematoma right ankle with oedema (n=1): not related
Vitamin B12 deficiency (n=1): not related
Numbness of the left side of the face (n=1): not related
Bursitis right elbow (n=1): not related
Skin infection leg (n=1): not related
Eczema (n=1): not related
**Moderate**
Insomnia (n=2): not related (n=1 leading to discontinuation)
Lumbar pain (n=2): not related
Arthrosis (n=2): not related (polyarthrosis of both hands n=1, gonarthrosis n=1)
Respiratory tract infection (n=2): not related (upper respiratory tract, bacterial n=1, bronchitis n=1)
Concentration difficulties (n=1): possibly related
Transient dysarthria (n=1): possibly related
Transient numbness of the face (n=1): possibly related
Intermittent falls (n=1): not related
Chronic widespread pain of the joints and muscles (n=1): not related
Chronic diarrhoea (n=1): not related
Autonomous reaction (n=1): not related
Nausea (n=1): not related
Vertebral fracture (thoracic vertebral body 7) associated with pain (n=1): not related
Osteopenia (n=1): not related
Worsening of PD (n=1): not related
Lipoma left arm and chest (n=1): not related
Restless legs syndrome (n=1): not related
**SAEs (n=2)**
**Moderate**
Worsening of PD (n=1): not related
**Severe, Life-threatening**
Adenocarcinoma of the rectum (n=1): not related
**Safety Follow-Up phase**
**AEs (n=7)**
**Mild**
Restless legs syndrome (n=1): not related
Intermittent panic attacks (n=1): not related
**Moderate**
Pain (n=3): not related (shoulder and knee n=1, diffuse neuropathic pain n=1, lumbar pain n=1)
Suspected borreliosis after bite of a tick (n=1): not related
Insomnia (n=1): not related
**SAEs (n=1)**
**Severe:**
Multiple herniated discs lumbar, degenerative changes in lumbar spine (n=1): not related

[a] Adverse events that started in the titration phase and continued in the open-label phase were noted here.
Abbreviations: OL, open-label; n, number; AE, adverse event; SAE, serious adverse event; PD, Parkinson´s Disease.

Supplementary Table 3: Change in endpoint scores during open-label administration of nabilone, patients n= 19

	V 1<br>n=21	V 3<br>n=19	Mean change between V 1 and V 3	p-value	Effect size
**MDS-UPDRS-I**	8.76 ±6.20 (8.00) (4.00 – 13.00)	9.47 ±5.90 (8.00) (4.00 – 14.00)	1.05 ±5.20 (-1.45; 3.56) (0.00, -1.00 – 5.00)	0.330	0.20
**MDS-UPDRS-II**	11.24 ±7.49 (11.00) (5.50 – 15.50)	11.11 ±6.82 (8.00) (6.00 – 16.00)	0.58 ±3.49 (-1.10; 2.26) (1.00, -1.00 – 3.00)	0.195	-0.17
**MDS-UPDRS-III**	27.86 ±11.97 (29.00) (18.00 – 37.50)	29.37 ±10.10 (28.00) (21.00 – 38.00)	1.89 ±6.88 (-1.42; 5.21) (1.00, -1.00 – 5.00)	0.092	-0.28
**MDS-UPDRS Total Score**	52.95 ±24.51 (50.00) (39.50 – 69.50)	52.58 ±22.45 (50.00) (37.00 – 58.00)	1.05 ±15.90 (-6.61; 8.71) (1.00, -4.00 – 10.00)	0.600	-0.07
**MDS-UPDRS Motor Sum Score II+III**	39.10 ±17.27 (40.00) (25.50 – 52.50)	40.47 ±15.36 (37.00) (30.00 – 46.00)	2.47 ±7.88 (-1.33; 6.27) (2.00, -2.00 – 8.00)	0.211	-0.31
**H&Y**	1.95 ±0.22 (2.00) (2.00 – 2.00)	2.11 ±0.32 (2.00) (2.00 – 2.00)	0.16 ±0.38 (-0.02; 0.34) (0.00, 0.00 – 0.00)	0.083	-0.42
**NMSS Domain 1**	1.57 ±1.86 (1.00) (0.00 – 2.00)	1.84 ±2.52 (1.00) (0.00 – 4.00)	0.63 ±2.75 (-0.70; 1.96) (0.00, -1.00 – 2.00)	0.502	-0.23
**NMSS Domain 2**	7.57 ±7.69 (5.00) (2.00 -10.50)	7.26 ±7.77 (5.00) (2.00 – 8.00)	0.16 ±6.36 (-2.91; 3.22) (-1.00, -3.00 – 3.00)	0.855	-0.03
**NMSS Domain 3**	4.52 ±5.03 (2.00) (0.00 – 8.00)	6.89 ±8.23 (4.00) (1.00 – 9.00)	2.37 ±7.34 (-1.17; 5.91) (0.00, -2.00 – 3.00)	0.461	-0.32
**NMSS Domain 4**	0.71 ±2.03 (0.00) (0.00 – 0.00)	0.16 ±0.50 (0.00) (0.00 – 0.00)	-0.63 ±2.01 (-1.60; 0.34) (0.00, 0.00 – 0.00)	0.180	0.32
**NMSS Domain 5**	3.67 ±6.39 (1.00) (0.00 – 5.00)	3.68 ±6.08 (2.00) (0.00 – 6.00)	0.47 ±2.09 (-0.53; 1.48) (0.00, 1.00 – 1.00)	0.375	-0.23
**NMSS Domain 6**	3.76 ±4.01 (3.00) (0.00 – 6.00)	3.74 ±4.57 (2.00) (0.00 – 8.00)	-0.05 ±3.91 (-1.94; 1.83) (0.00, -3.00 – 2.00)	0.850	0.01
**NMSS Domain 7**	6.24 ±6.48 (4.00) (0.00 – 12.50)	6.74 ±5.76 (6.00) (2.00 – 13.00)	0.47 ±4.06 (-1.48; 2.43) (0.00, -2.00 – 4.00)	0.598	-0.12
**NMSS Domain 8**	0.00 ±0.00 (0.00) (0.00 – 0.00)	0.42 ±1.12 (0.00) (0.00 – 0.00)	0.42 ±1.12 (-0.12; 0.96) (0.00, 0.00 – 0.00)	0.109	-0.38
**NMSS Domain 9**	7.57 ±5.60 (7.00) (4.00 – 12.00)	8.21 ±5.90 (8.00) (3.00 – 12.00)	1.00 ±4.91 (-1.37; 3.37) (1.00, -1.00 – 4.00)	0.324	-0.20
**NMSS Total Score**	35.62 ±25.69 (33.00) (14.00 – 52.00)	38.95 ±25.82 (29.00) (18.00 – 62.00)	4.84 ±18.08 (-3.87; 13.55) (2.00, -8.00 – 15.00)	0.212	-0.27
**KPPS Total Score**	6.95 ±8.29 (4.00) (0.50 – 12.00)	13.42 ±13.60 (8.00) (1.00 – 24.00)	6.84 ±15.12 (-0.45; 14.13) (4.00, -1.00 – 20.00)	0.073	-0.45
**HADS-A**	4.57 ±3.01 (4.00) (2.00 – 7.00)	4.68 ±3.74 (4.00) (2.00 – 9.00)	0.16 ±1.50 (-0.57; 0.88) (0.00, -1.00 – 2.00)	0.560	-0.11

**HADS-D**	5.43 ±4.07 (5.00) (1.50 – 9.00)	4.42 ±3.20 (4.00) (2.00 – 7.00)	-1.00 ±2.08 (-2.00; 0.00) (0.00, -3.00 – 0.00)	0.044	0.48
**PDQ-8 SI**	51.94 ±16.64 (56.25) (34.38 – 64.06)	54.11 ±16.44 (56.25) (40.63 – 68.75)	2.96 ±9.11 (-1.43; 7.35) (3.13, -3.13 – 6.25)	0.236	-0.33
**ESS**	8.14 ±5.79 (6.00) (3.50 – 13.00)	7.32 ±4.58 (7.00) (4.00 – 8.00)	-0.11 ±2.75 (-1.43; 1.22) (0.00, -1.00 – 2.00)	0.886	0.04
**FSS**	34.62 ±14.71 (36.00) (26.00 – 45.50)	29.89 ±14.35 (27.00) (19.00 – 39.00)	-4.26 ±10.08 (-9.12; 0.60) (-2.00, -10.00 – 0.00)	0.103	0.42
**QUIP RS**	0.48 ±1.12 (0.00) (0.00 – 0.00)	0.37 ±1.12 (0.00) (0.00 – 0.00)	-0.11 ±1.41 (-0.78; 0.57) (0.00, 0.00 – 0.00)	0.785	0.08

Data are given as mean ± standard deviation (95% confidence interval) (median, interquartile range / P25-P75).
Abbreviations: CI, confidence interval; MDS-UPDRS, Movement Disorder Society- Unified Parkinson´s Disease Rating Scale; NMSS, Non-Motor Symptoms Scale; CGI-I, Clinical Global Impression – Global Improvement; KPPS, King's PD pain scale; HADS-A/-D, Hospital anxiety and depression scale - Anxiety/ - Depression; PDQ-8 SI, Parkinson´s Disease Questionnaire – 8 Summary Index; ESS, Epworth Sleepiness Scale; FSS, Fatigue Severity Scale; QUIP-RS, Questionnaire for Impulsive-Compulsive Disorders in Parkinson’s Disease–Rating Scale. Higher score values indicate worse outcome in all scales and questionnaires.
NMSS Domains: Domain 1: Cardiovascular, Domain 2: Sleep/Fatigue, Domain 3: Mood/Apathy, Domain 4: Perceptual problems/Hallucinations, Domain 5: Attention/Memory. Domain 6, Domain 7: Urinary, Domain 8: Sexual dysfunction, Domain 9: Miscellaneous.
For all p-values, significance level was set at p≤0.05. Effect size according to Cohen´s D for all variables except for the CGI-I (ϕ coefficient). Cohen´s D of 0.2, 0.5, and 0.8 and ϕ coefficient of 0.1, 0.3, and 0.5 were considered ‘small’, ‘medium’, and ‘large’ effect sizes.

Supplementary Table 4: Differences between the former nabilone and placebo groups in relevant efficacy outcomes

	Group assigned from the NMS-Nab1 Study [a]	SCR (n=21)	V3 (n=19)	Between-group difference (95% CI)	p-value [b]	Effect size [b]
**MDS-UPDRS-I**	P	11.10 ±4.33 (10.50) (7.50 – 13.50)	8.67 ±5.15 (8.00) (4.00 – 12.50)	2.36 (-3.88; 6.08)	0.647	0.11
	N	12.91 ±6.96 (11.00) (9.00 – 18.00)	10.20 ±6.70 (10.50) (2.75 – 15.00)			
**MDS-UPDRS item 1.7** [c]	P	2.50 ±0.97 (2.00) (2.00 – 3.25)	0.78 ±0.83 (1.00) (0.00 – 1.50)	-0.47 (-1.96; 1.03)	0.514	0.18
	N	2.55 ±1.13 (2.00) (2.00 – 4.00)	1.40 ±1.35 (1.00) (0.00 – 2.25)			
**MDS-UPDRS item 1.9** [c]	P	2.30 ±0.48 (2.00) (2.00 – 3.00)	1.67 ±0.71 (2.00) (1.00 – 2.00)	0.13 (-0.85; 1.12)	0.779	0.07
	N	2.09 ±0.70 (2.00) (2.00 – 3.00)	1.30 ±1.34 (1.00) (0.00 – 2.25)			
**NMSS Domain 2** [c]	P	16.40 v12.31 (13.50) (6.00 – 24.50)	5.44 ±5.64 (5.00) (1.00 -8.00)	-4.36 (5.35; -15.64)	0.427	0.19
	N	14.64 ±12.00 (12.00) (6.00 – 20.00)	8.90 ±9.28 (5.00) (2.75 – 15.75)			
**KPPS Total Score**	P	20.00 ±14.33 (16.00) (9.75 – 29.25)	14.67 ±12.24 (16.00) (3.50 – 24.00)	3.17 (-11.34; 17.67)	0.651	0.11
	N	21.64 ±13.68 (17.00) (9.00 -32.00)	12.30 ±15.28 (8.00) (0.75 – 17.50)			

Data of continuous variables are presented as mean ± standard deviation (median, P25-P75) or mean (95% CI).
Abbreviations: CI, confidence interval; MDS-UPDRS, Movement Disorder Society- Unified Parkinson´s Disease Rating Scale; NMSS, Non-Motor Symptoms Scale; KPPS, King's Parkinson´s Disease pain scale; SCR, screening; V, visit.
Higher Score values indicate worse outcome in all scales and questionnaires.
[a] This column refers to the randomized double-blind group assignment in the previous NMS-Nab1 study. N refers to former nabilone-group and P to the placebo-group. 11 patients at SCR and 10 patients at V3 were in the former nabilone group. 10 patients at SCR and 9 patients at V3 were in the former placebo group.
For all p-values, significance level was set at p≤0.05. Student´s t-test. [b] refers to the difference between the former nabilone and placebo group. Effect size according to Cohen´s D. Cohen´s D of 0.2, 0.5, and 0.8 were considered 'small', 'medium', and 'large' effect sizes.
[c] MDS-UPDRS-1.7: Nighttime sleep problems, 1.9: Pain and other sensations; NMSS Domain 2: Sleep/Fatigue.
